# Expression of SPRR3 is associated with tumor cell proliferation and invasion in glioblastoma multiforme

**DOI:** 10.3892/ol.2013.1736

**Published:** 2013-12-06

**Authors:** QINGYANG LIU, CHUANBAO ZHANG, GUOFO MA, QUANGENG ZHANG

**Affiliations:** 1 Department of Immunology, Institute of Basic Medical Sciences, Capital Medical University, Beijing 100069, P.R. China; 2 Department of Neurosurgery, Tiantan Hospital, Capital Medical University, Beijing 100050, P.R. China

**Keywords:** glioblastoma, SPRR3, cellular proliferation, invasion, methylation profiling

## Abstract

Esophagin, also known as small proline-rich protein 3 (SPRR3), has been demonstrated to be important in the initiation and progression of numerous types of tumor, including colorectal and breast cancer. However, studies concerning the biological functions of SPRR3 in glioblastoma multiforme (GBM) are limited. Therefore, we aimed to identify the functions and molecular mechanisms underlying the role of SPRR3 in GBM. Hypomethylation of SPRR3 was observed and associated with a poor clinical outcome in GBM patients compared with healthy individuals by using gene methylation profiling. The present study was performed to investigate the expression status and effects of SPRR3 in GBM. The U251 cell line was used in the functional analyses. Cell growth was examined by MTT and colony formation assay. Cell invasion was measured using the Transwell invasion assay. The expression of SPRR3 in tissue samples was examined by immunohistochemistry. The results revealed that the overexpression of SPRR3 accelerates U251 cell proliferation and invasion. It was also observed that SPRR3 was markedly upregulated in 72.7% of GBM samples (24/33) compared with the normal tissue. These results suggest that an increased expression of SPRR3 is involved in tumorigenesis.

## Introduction

Glioblastoma multiforme (GBM) is the most common primary brain tumor in adults and is associated with the highest rate of mortality (1). Numerous biological processes occur in tumorigenesis. CpG island hypermethylation silences tumor suppressor genes, whereas hypomethylation promotes the transcriptional activation of oncogenes and induces chromosomal instability (2,3). Several aberrantly methylated genes have been reported in gliomas. For instance, the p53/Mdm2/p14ARF cell cycle control pathway genes are affected by CpG island promoter hypermethylation (4).

Gene methylation profiling is able to simultaneously evaluate thousands of individual gene methylation levels and reveal molecular signatures that reflect potential pathogenic mechanisms and are associated with survival. In the present study, we performed genome-wide gene methylation level analysis and identified SPRR3 as a candidate gene, which showed aberrant levels between GBM patients and healthy individuals.

The small proline-rich proteins (SPRRs) are encoded by a multigene family clustered within the epidermal differentiation complex on human chromosome 1q21 (5–7). SPRR proteins are known to be markers for terminal squamous cell differentiation; however, they also function in nonsquamous tissues (8). SPRR3 (also named esophagin) is abundantly expressed in oral and esophageal epithelia (9,10). SPRR3 has been considered as a differentiation marker of squamous epithelium, since its expression is strictly correlated with keratinocyte terminal differentiation (5,11). SPRR3 is frequently downregulated in esophageal squamous cell carcinoma (ESCC) and it has been demonstrated to suppress the tumorigenicity of ESCC cells (4,7,12). However, it has previously been reported that SPRR3 is upregulated in colorectal and breast cancer (13,14), suggesting that SPRR3 is associated with malignant tumorigenesis.

In the present study, we found that SPRR3 hypomethylation was associated with the clinical outcome in GBM patients. U251 cells demonstrated a significant decrease in proliferation and invasion following specific knockdown of SPRR3. Immunohistochemical staining demonstrated that SPRR3 was highly expressed in tumors compared with normal tissue.

## Materials and methods

### Patients, tissue samples and cell lines

All glioma samples included in the present study were obtained from the Chinese Glioma Genome Atlas (http://www.cgga.org.cn). The patients underwent surgical resection between January 2006 and December 2010, and subsequently received radiation therapy or concomitant and adjuvant temozolomide chemotherapy. Tumor tissue samples were obtained by surgical resection provided that the diagnosis of glioma was established according to the 2007 WHO classification, and eight normal brain tissues were included. The present study was approved by the institutional review boards of Beijing Tiantan Hospital (Beijing, China) and written informed consent was obtained from all patients. U251 glioma cells were purchased from the Chinese Academy of Sciences Cell Bank (Kunming, Yunnan, China). U251 cells were cultured in Dulbecco’s modified Eagle’s medium (DMEM) supplemented with 10% fetal bovine serum (FBS). All cells were maintained in a 37°C, 5% CO_2_ incubator and routinely passaged at 2–3 day intervals.

### Genomic DNA extraction and DNA methylation profiling

All the tissue samples were immediately snap-frozen in liquid nitrogen following surgery. Genomic DNA from frozen tumor tissues was extracted using the QIAamp DNA Mini kit (Qiagen, Hilden, Germany) according to the manufacturer’s instructions. DNA concentrations were measured using a NanoDrop ND-1000 spectrophotometer (NanoDrop Technologies, Houston, TX, USA). Illumina Infinium HumanMethylation27 BeadChips (Illumina Inc., San Diego, CA, USA) were used as previously described (15). Methylation of 27,578 CpG sites at 14,475 consensus coding sequencing sites was performed following the manufacturer’s instructions at the Wellcome Trust Centre for the Human Genetics Genomics Lab (Oxford, UK). The array results were analyzed with the BeadStudio software (Illumina Inc.).

### SPRR3 gene knockdown by siRNA

Logarithmically growing cells were seeded at a density of 10^5^ cells per 6-cm dish. Oligonucleotide transfection was performed using the siRNA and siRNA negative control (NC; Table I) that were chemically synthesized by the Shanghai GenePharma Company (Shanghai, China). Cells were transfected using Lipofectamine 2000 reagent (Invitrogen Life Technologies, Carlsbad, CA, USA). Transfection complexes were prepared according to the manufacturer’s instructions and added directly to the glioma cells, resulting in a final oligonucleotide concentration of 10 nmol/l. The transfection medium was replaced 6 h post-transfection. Cells were used for *in vitro* functional assay.

### Western blot analysis

Following cell treatment, in order to determine the levels of SPRR3 expression, total protein was isolated in lysis buffer. Equal amounts of protein (15 μg) were loaded into the sample wells and separated on a 10% SDS-polyacrylamide gel, and transferred onto polyvinylidene difluoride membranes. Immunoblot analysis was performed with the mouse antibodies against SPRR3 (ab58233; Abcam, Hong Kong, China; 1:1,000 dilution). β-actin (anti-β-actin antibody was obtained from Proteintech Group, Chicago, IL, USA; 1:4,000 dilution) was reblotted to check for equal loading of the gel.

### MTT and colony formation assay

The MTT assay was used to determine relative cell growth. U251 cells were plated at a density of 5,000 cells per well 24 h after transfection with siRNA in 96-well plates with six replicate wells for each condition. For quantitation of cell viability, cultures were stained after 5 days. A cell growth assay was performed using MTT (thiazolyl blue). In brief, 20 μl of 5 mg/ml MTT solution was added to each well and incubated for 4 h at 37°C. The cell viability was determined at an absorbance of 490 nm following solubilization in 150 μl DMSO. All data points represent the mean of a minimum of six wells. A colony formation assay was performed and briefly U251 cells were transfected with siRNA or the NC for 48 h, and seeded into the individual wells of a six-orifice plate (2,000 per orifice). Following culture for 14 days, all orifices were washed with PBS and stained with crystal violet. The number of colonies with >30 cells was counted. The colonies were manually counted using a microscope (Leica DM6000 B; Upright Microscopes, Wetzlar, Germany).

### Transwell invasion assay

Cell culture chambers (24-well) with Transwell inserts (Corning Life Sciences, Corning, NY, USA) with an 8-μm pore membrane precoated with Matrigel (BD Biosciences, San Jose, CA, USA) were used in the Transwell invasion assay. U251 cells were plated at a density of 1×10^4^ per upper well in 200 μl culture medium (DMEM, without FBS) and the lower chamber was filled with 500 μl of medium (DMEM, 10% FBS) in the set control group, siRNA control group and siRNA group. The cells were incubated at 37°C and allowed to invade for 24 h, following which, the upper surface of the membrane of the non-invading cells was removed by scrubbing with a cotton-tipped swab. Cells on the lower surface of the filter were fixed for 20 min in absolute ethyl alcohol and stained with crystal violet after being air-dried briefly. The mean number of invaded cells was counted from five preselected microscopic fields at magnification ×200 and all experiments were performed in triplicate.

### Immunohistochemistry

Surgical specimens were fixed in formalin, routinely processed and paraffin embedded, then cut into 4-μm sections, deparaffinized with xylene and rehydrated. Sections were submerged in EDTA (pH 8.0), autoclaved for antigen retrieval and then treated with 3% hydrogen peroxide, followed by incubation with 1% FBS. Anti-SPRR3 antibody (Abcam; mouse monoclonal; 1:200 dilution) was added as a primary antibody and incubated at 4°C for 2 h. Normal mouse serum was used for the NC and horseradish peroxidase-labeled secondary antibody (Santa Cruz Biotechnology, Inc., Santa Cruz, CA, USA) was applied and incubated for 45 min at 37°C, followed by 5 min incubation at room temperature with DAB for color development. Finally, the sections were counterstained with hematoxylin and mounted with Permount (BIOS, Beijing, China). The immunohistochemical staining results were visualized and images were captured under a bright-field microscope (Olympus BX-51; Olympus Optical Co., Ltd., Tokyo, Japan). The SPRR3 cytoplasmic expression was classified into two categories determined by combining the proportion of positively stained tumor cells and the intensity of staining. The intensity of staining was scored by two investigators without knowledge of clinical information on a scale of 0 to 3 (0, negative; 1, slightly positive; 2, moderately positive; 3, intensely positive). A score of 0 and 1 or 2 and 3 indicated low or high expression, respectively.

### Statistical and bioinformatics analysis

Kaplan-Meier survival analysis was used to estimate the survival distributions and the overall survival (OS) time was calculated from the date of diagnosis until mortality or the last follow-up contact. Significant differences among the groups were determined using Student’s t-test. All analyses were two-tailed. P<0.05 was considered to indicate a statistically significant difference. Analyses were performed using Matlab 2009b (MathWorks, Natick, MA, USA), GraphPad Prism (GraphPad Software Inc., La Jolla, CA, USA) and SPSS version 13.0 (SPSS, Inc., Chicago, IL, USA).

## Results

### DNA methylation profile analysis

**Figure 1:**
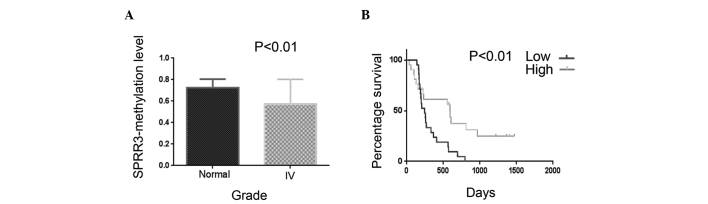
Methylation level of SPRR3 in gliomas and association with survival in glioblastoma multiforme. (A) Methylation levels of SPRR3 in healthy individuals and high-grade gliomas, analyzed by DNA methylation profiling containing 227 frozen glioma tissues. The methylation level of SPRR3 was significantly lower in high-grage glioma patients compared with those in healthy individuals (P<0.001). (B) Kaplan-Meier survival curves according to the methylation level of SPRR3 in 42 frozen high-grade glioma tissues. The log-rank test was used to calculate P-values. SPRR3, small proline-rich protein 3.

To investigate the molecular changes associated with GBM tumorigenesis, we analyzed the gene methylation level in 42 patients with GBM and eight healthy individuals using a DNA methylation profile (Table II). The methylation levels of 14,475 genes were analyzed using t-tests. Among these genes, SPRR3 was demonstrated to be associated with molecular changes and its methylation level was significantly lower in GBM patients compared with healthy individuals (Fig. 1). The OS time was measured through Kaplan-Meier survival curve analysis and the results revealed that glioma patients with a low methylation level of SPRR3 had significantly lower progression-free survival rates (P=0.010) and OS times (P=0.007) than patients with a high methylation level. Kaplan-Meier survival curves, according to the methylation level of SPRR3 in 42 frozen glioma tissues, demonstrated that SPRR3 hypomethylation was associated with a poor clinical outcome in GBM patients (Fig. 1). These results indicated that the methylation level of SPRR3 is an independent prognostic marker in glioma patients.

### SPRR3 is upregulated in glioblastoma

**Figure 2:**
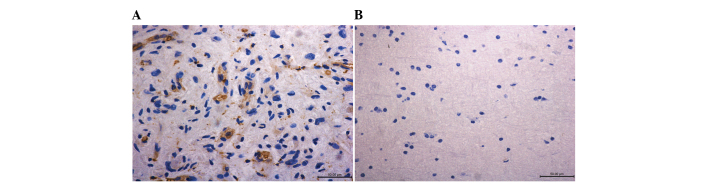
SPRR3 overexpression in glioma. Immunohistochemical staining demonstrated that SPRR3 was highly expressed in glioblastoma multiforme compared with the normal tissue (magnification, ×400). (A) Glioblastoma (WHO grade IV); (B) normal tissue of the brain. SPRR3 was expressed in stroma. SPRR3, small proline-rich protein 3.

Samples of the tumor immunohistochemical staining demonstrated that SPRR3 was highly expressed in tumors compared with the normal tissue (Fig. 2). The quantification of immunohistochemical signals for 33 GBM samples revealed that 24 of the samples demonstrated positive staining for SPRR3 (72.7%) while only 11.1% of the normal tissue stained positively for SPRR3 (one in nine samples). No significant differences in patient age and gender were identified (Table III). We also measured the OS time according to SPRR3 expression levels in glioma patients through Kaplan-Meier survival estimates; however, the results revealed that there was no significant correlation between the two subgroups with high or low SPRR3 expression (high expression, ≥2; low expression, <2).

### Identification of SPRR3-associated proliferation and invasion signature in GBM

**Figure 3:**
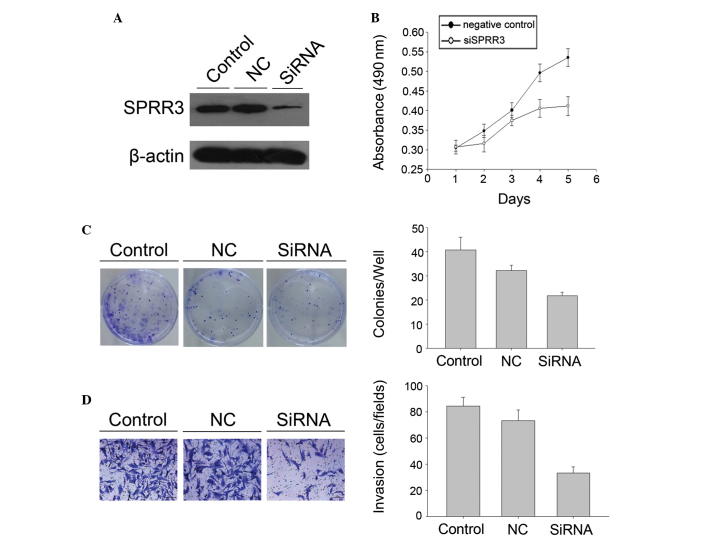
SPRR3 promotes the proliferation and invasion of U251 cell lines. (A) Expression of SPRR3 was significantly reduced following knockdown by siRNA. β-actin was used as the control. (B) Proliferation of U251 cell lines was assessed by MTT assay and a decrease in absorbance was observed following the knockdown of SPRR3. (C) Number of colonies formed by cells treated with siRNA of SPRR3 was decreased compared with the control cells (P<0.05). (D) Invasion of the U251 cell line assessed by Transwell assay (magnification, ×200). The number of invading cells was significantly less in the siRNA group compared with the control and siRNA control groups (P<0.05). NC, negative control; SPRR3, small proline-rich protein 3.

As the results of the immunohistochemical staining demonstrated that SPRR3 was highly expressed in GBM samples, we investigated the effect of SPRR3 on the proliferation and invasion of GBM cells. SPRR3 siRNA was used to specifically knock down SPRR3 expression in U251 cell lines (Fig. 3). The MTT assay demonstrated a significant decrease in proliferation in siRNA U251 cell lines compared with the cells transfected with the control (Fig. 3). The same result was found in the colony formation assay and the siRNA U251 cell lines demonstrated a decrease in focus numbers compared with the control (P<0.05; Fig. 3). Cell invasion was measured using the Transwell invasion assay and the results demonstrated that U251 cell lines significantly attenuated invasiveness following SPRR3 knockdown, as indicated by a marked decrease in the number of invaded cells (P<0.05; Fig. 3).

## Discussion

SPRR3 promoted GBM cell (U251) proliferation and invasion. The results of the immunohistochemical staining demonstrated that SPRR3 was upregulated in the tissue samples of more than half of the GBM patients (72.7%) and the methylation level of SPRR3 was correlated with the clinical outcome in glioma patients validated by DNA methylation profile analysis. The present study, to the best of our knowledge, is the first to examine the functions and methylation level of SPRR3 in GBM.

GBM is the most common and aggressive primary brain tumor in adults. Its prognosis remains extremely poor, despite multimodal treatment by surgery, radiotherapy and chemotherapy (16). Aberrations in DNA methylation patterns may have critical effects on tumor initiation and progression (17). DNA hypermethylation of specific genes and DNA hypomethylation commonly affecting repetitive DNA are observed in brain cancers (18–24).

In order to investigate the potential molecular mechanisms underlying GBM tumorigenesis, we analyzed gene methylation levels in 227 patients with glioma (141 low-grade gliomas, 44 anaplastic gliomas and 42 GBMs) and eight healthy individuals by using a DNA methylation profile. We found that the methylation levels of several genes were significantly altered (SPRR3, TES, RGN, FAM12B, AJAP1, PDE4C and SPRR2D). Among these genes, we found that SPRR3 hypomethylation was a common event in the majority of cases, regardless of the sex and age of the patients, and SPRR3 hypomethylation was also demonstrated to be associated with an adverse prognosis. In the present study, we found that SPRR3 was hypomethylated in GBM. Previously, Ammerpohl *et al*(25) demonstrated the same result in cirrhotic liver and hepatocellular carcinoma. Mayol *et al* also found that SPRR3 hypomethylation affected cancer-related biological functions and genes relevant to neuroblastoma pathogenesis (26).

SPRR3 is frequently downregulated in ESCC, where it is known to inhibit tumorigenesis. Notably, in contrast to the findings in ESCC, in the present study, SPRR3 promoted GBM cell (U251) proliferation and invasion, and samples from the tumor immunohistochemical staining demonstrated that SPRR3 was highly expressed in the majority of tumors compared with the normal tissue. Previously, SPRR3 was demonstrated to be upregulated in colorectal and breast cancer, and these results were in accordance with our findings. Our study suggests that upregulation of SPRR3 occurs in GBM tumorigenesis.

Considering the vital role of cellular proliferation and invasion in GBM pathogenesis, we investigated the effect of the overexpression of SPRR3 on GBM cell lines. SPRR3 siRNA was used to specifically knock down SPRR3 expression. The MTT assay demonstrated a significant decrease in proliferation and was consistent with the colony formation assay results in the U251 glioma cell line following transfection (Fig. 3). Cellular proliferation is one of the most important biological processes in tumorigenesis due to its role in growth and in the maintenance of tissue homeostasis (27,28). Each type of human cancer often has specific signaling pathways to rely on for cellular proliferation (29). In colorectal cancer, in which SPRR3 was upregulated and accelerated cell proliferation, it was proposed that the effect of SPRR3 in promoting colorectal tumorigenesis is associated with the degradation of p53 caused by AKT activation (13). The same results were also found in breast cancer. SPRR3 has been demonstrated to promote breast cancer cell proliferation by enhancing p53 degradation via the AKT and MAPK pathways (14). We used a similar experiment and demonstrated the same results in the U251 glioma cell line. AKT signaling has been implicated in angiogenesis, the promotion of tumor invasion as well as in tumor cell survival (30,31). In particular, a higher proliferation index of the tumor cells has been associated with shorter survival rates (32,33). As AKT signaling promotes tumor invasion, we used a Transwell invasion assay to measure cell invasiveness and the results demonstrated that the invasiveness of U251 cell lines significantly decreased following the knock down of SPRR3 expression.

The present study demonstrated that SPRR3 is hypomethylated and upregulated in glioblastoma, and the integrated microarray analysis revealed that SPRR3 hypomethylation was associated with an adverse prognosis. We also demonstrated that the overexpression of SPRR3 may be associated with cell proliferation and invasion in GBM, and that it may be a candidate biomarker for GBM.

## Figures and Tables

**Table I:** siRNA and negative control strand sequences.

	Sense	Antisense
siRNA	GCCAUAGUCUCUCUCUUAUTT	AUAAGAGAGAGACUAUGGCTT
Negative control	UUCUCCGAACGUGUCACGUTT	ACGUGACACGUUCGGAGAATT

siRNA strand was from 5′ to 3′.

**Table II:** Variables associated with the methylation level of SPRR3 in 42 glioma samples.

			SPRR3 methylation level		
				
Variable	No. of patients	Median OS (days)	Low	High	P-value
Gender					0.617
Male	26	261	4	22	
Female	16	241	3	13	
Age (years)					0.545
≤50	28	348	7	21	
>50	14	211	0	14	

OS, overall survival; SPRR3, small proline-rich protein 3.

**Table III:** Variables associated with the expression of SPRR3 in 34 glioma samples.

			SPRR3 expression level		
				
Variable	No. of patients	Median OS (days)	Low	High	P-value
Gender					0.935
Male	23	447	5	18	
Female	11	315	2	9	
Age (years)					0.798
≤50	14	532.5	2	12	
>50	20	315	5	15	

OS, overall survival; SPRR3, small proline-rich protein 3.
